# Supplementary material for: The epidemiology of soil-transmitted helminth infections in children up to 8 years of age: Findings from an Ecuadorian birth cohort
Source: PLoS Negl Trop Dis. 2021 Nov 19;15(11):e0009972. doi: 10.1371/journal.pntd.0009972 (PMC8641893; doi:10.1371/journal.pntd.0009972)
Supplement: S1 Table — Shown are estimated proportions (prop) (and 95% confidence intervals [CI]) infected by age using two-level logistic regression accounting for the household hierarchical structure of the data. S. stercoralis–Strongyloides stercoralis. (DOCX) [file pntd.0009972.s001.docx]

|  | **Any STH** | | | ***A. lumbricoides*** | | | ***T. trichiura*** | | | **Hookworm** | ***S. stercoralis*** |
| --- | --- | --- | --- | --- | --- | --- | --- | --- | --- | --- | --- |
| **AGE (years)** | **Prop** | **Lower 95% CI** | **Upper**  **95% CI** | **Prop** | **Lower**  **95% CI** | **Upper**  **95% CI** | **Prop** | **Lower 95% CI** | **Upper**  **95% CI** | **Prop** | **Prop** |
| <1 | 0.198 | 0.162 | 0.235 | 0.088 | 0.065 | 0.111 | 0.073 | 0.052 | 0.095 | 0.002 | 0.001 |
| 1 | 0.211 | 0.175 | 0.247 | 0.093 | 0.070 | 0.115 | 0.083 | 0.061 | 0.105 | 0.003 | 0.002 |
| 2 | 0.242 | 0.208 | 0.275 | 0.103 | 0.082 | 0.125 | 0.110 | 0.087 | 0.133 | 0.003 | 0.002 |
| 3 | 0.271 | 0.239 | 0.303 | 0.113 | 0.093 | 0.134 | 0.138 | 0.114 | 0.161 | 0.004 | 0.002 |
| 4 | 0.299 | 0.268 | 0.329 | 0.123 | 0.103 | 0.143 | 0.166 | 0.141 | 0.190 | 0.004 | 0.002 |
| 5 | 0.324 | 0.295 | 0.353 | 0.132 | 0.112 | 0.152 | 0.192 | 0.167 | 0.217 | 0.005 | 0.003 |
| 6 | 0.346 | 0.317 | 0.374 | 0.140 | 0.120 | 0.160 | 0.215 | 0.189 | 0.242 | 0.006 | 0.003 |
| 7 | 0.365 | 0.337 | 0.394 | 0.147 | 0.127 | 0.167 | 0.235 | 0.207 | 0.263 | 0.007 | 0.003 |
| 8 | 0.381 | 0.352 | 0.410 | 0.153 | 0.132 | 0.174 | 0.250 | 0.221 | 0.280 | 0.008 | 0.003 |
| 9 | 0.394 | 0.364 | 0.423 | 0.158 | 0.136 | 0.179 | 0.262 | 0.231 | 0.292 | 0.009 | 0.004 |
| 10 | 0.404 | 0.374 | 0.434 | 0.161 | 0.140 | 0.183 | 0.269 | 0.238 | 0.300 | 0.010 | 0.004 |
| 11 | 0.411 | 0.380 | 0.441 | 0.164 | 0.142 | 0.186 | 0.272 | 0.241 | 0.303 | 0.011 | 0.004 |
| 12 | 0.415 | 0.384 | 0.446 | 0.166 | 0.143 | 0.188 | 0.271 | 0.241 | 0.302 | 0.012 | 0.004 |
| 13 | 0.417 | 0.386 | 0.447 | 0.166 | 0.144 | 0.189 | 0.268 | 0.238 | 0.297 | 0.014 | 0.005 |
| 14 | 0.416 | 0.386 | 0.446 | 0.166 | 0.144 | 0.188 | 0.261 | 0.233 | 0.290 | 0.015 | 0.005 |
| 15 | 0.413 | 0.383 | 0.443 | 0.165 | 0.143 | 0.187 | 0.253 | 0.225 | 0.281 | 0.016 | 0.005 |
| 16 | 0.409 | 0.379 | 0.438 | 0.163 | 0.142 | 0.185 | 0.243 | 0.217 | 0.270 | 0.017 | 0.005 |
| 17 | 0.402 | 0.373 | 0.431 | 0.161 | 0.140 | 0.181 | 0.232 | 0.207 | 0.258 | 0.017 | 0.005 |
| 18 | 0.394 | 0.366 | 0.423 | 0.157 | 0.137 | 0.178 | 0.220 | 0.196 | 0.245 | 0.018 | 0.005 |
| 19 | 0.385 | 0.358 | 0.413 | 0.154 | 0.134 | 0.174 | 0.208 | 0.185 | 0.232 | 0.019 | 0.005 |
| 20 | 0.375 | 0.348 | 0.402 | 0.150 | 0.131 | 0.169 | 0.196 | 0.173 | 0.219 | 0.020 | 0.006 |
| 21 | 0.364 | 0.337 | 0.390 | 0.146 | 0.127 | 0.164 | 0.184 | 0.162 | 0.206 | 0.020 | 0.006 |
| 22 | 0.352 | 0.326 | 0.378 | 0.141 | 0.123 | 0.160 | 0.172 | 0.151 | 0.193 | 0.020 | 0.006 |
| 23 | 0.340 | 0.314 | 0.366 | 0.137 | 0.119 | 0.155 | 0.161 | 0.141 | 0.182 | 0.021 | 0.006 |
| 24 | 0.327 | 0.302 | 0.353 | 0.132 | 0.115 | 0.150 | 0.151 | 0.131 | 0.170 | 0.021 | 0.005 |
| 25 | 0.315 | 0.289 | 0.340 | 0.127 | 0.110 | 0.145 | 0.141 | 0.122 | 0.160 | 0.021 | 0.005 |
| 26 | 0.302 | 0.277 | 0.327 | 0.123 | 0.106 | 0.140 | 0.132 | 0.114 | 0.151 | 0.020 | 0.005 |
| 27 | 0.289 | 0.264 | 0.314 | 0.118 | 0.101 | 0.135 | 0.124 | 0.107 | 0.142 | 0.020 | 0.005 |
| 28 | 0.277 | 0.252 | 0.302 | 0.114 | 0.097 | 0.130 | 0.117 | 0.100 | 0.134 | 0.020 | 0.005 |
| 29 | 0.265 | 0.240 | 0.290 | 0.109 | 0.093 | 0.126 | 0.110 | 0.094 | 0.127 | 0.019 | 0.005 |
| 30 | 0.253 | 0.229 | 0.278 | 0.105 | 0.089 | 0.121 | 0.105 | 0.089 | 0.121 | 0.019 | 0.005 |
| 31 | 0.242 | 0.218 | 0.267 | 0.101 | 0.085 | 0.117 | 0.099 | 0.084 | 0.115 | 0.018 | 0.005 |
| 32 | 0.232 | 0.207 | 0.256 | 0.097 | 0.082 | 0.113 | 0.095 | 0.080 | 0.110 | 0.018 | 0.005 |
| 33 | 0.222 | 0.197 | 0.246 | 0.094 | 0.078 | 0.110 | 0.091 | 0.076 | 0.106 | 0.017 | 0.004 |
| 34 | 0.213 | 0.188 | 0.237 | 0.091 | 0.075 | 0.106 | 0.088 | 0.073 | 0.102 | 0.016 | 0.004 |
| 35 | 0.204 | 0.180 | 0.228 | 0.087 | 0.072 | 0.103 | 0.085 | 0.070 | 0.099 | 0.016 | 0.004 |
| 36 | 0.196 | 0.172 | 0.220 | 0.085 | 0.069 | 0.100 | 0.082 | 0.068 | 0.097 | 0.015 | 0.004 |
| 37 | 0.189 | 0.165 | 0.213 | 0.082 | 0.067 | 0.097 | 0.080 | 0.066 | 0.095 | 0.014 | 0.004 |
| 38 | 0.183 | 0.159 | 0.206 | 0.079 | 0.064 | 0.094 | 0.079 | 0.064 | 0.094 | 0.014 | 0.004 |
| 39 | 0.177 | 0.153 | 0.200 | 0.077 | 0.062 | 0.092 | 0.078 | 0.062 | 0.093 | 0.013 | 0.003 |
| 40 | 0.171 | 0.148 | 0.195 | 0.075 | 0.060 | 0.090 | 0.077 | 0.061 | 0.092 | 0.012 | 0.003 |
| 41 | 0.167 | 0.143 | 0.190 | 0.073 | 0.059 | 0.088 | 0.076 | 0.060 | 0.092 | 0.011 | 0.003 |
| 42 | 0.163 | 0.139 | 0.186 | 0.072 | 0.057 | 0.086 | 0.076 | 0.059 | 0.092 | 0.011 | 0.003 |
| 43 | 0.159 | 0.136 | 0.183 | 0.070 | 0.055 | 0.085 | 0.076 | 0.059 | 0.093 | 0.010 | 0.003 |
| 44 | 0.156 | 0.133 | 0.180 | 0.069 | 0.054 | 0.084 | 0.076 | 0.058 | 0.093 | 0.010 | 0.003 |
| 45 | 0.154 | 0.130 | 0.178 | 0.068 | 0.053 | 0.083 | 0.076 | 0.058 | 0.094 | 0.009 | 0.003 |
| 46 | 0.152 | 0.127 | 0.177 | 0.067 | 0.051 | 0.082 | 0.076 | 0.057 | 0.095 | 0.008 | 0.002 |
| 47 | 0.151 | 0.126 | 0.176 | 0.066 | 0.050 | 0.082 | 0.077 | 0.057 | 0.096 | 0.008 | 0.002 |
| 48 | 0.150 | 0.124 | 0.176 | 0.065 | 0.049 | 0.081 | 0.078 | 0.057 | 0.098 | 0.007 | 0.002 |
| 49 | 0.150 | 0.123 | 0.177 | 0.065 | 0.048 | 0.081 | 0.078 | 0.057 | 0.099 | 0.007 | 0.002 |
| 50 | 0.150 | 0.122 | 0.178 | 0.064 | 0.047 | 0.082 | 0.079 | 0.057 | 0.101 | 0.007 | 0.002 |

S1 Table. Estimated risk of infection with soil-transmitted helminths (STH) using data from stool samples collected from 6,800 individuals living 1,973 cohort households around the time of birth of the cohort child. Shown are estimated proportions (prop) (and 95% confidence intervals [CI]) infected by age using two-level logistic regression accounting for the household hierarchical structure of the data. *S. stercoralis – Strongyloides stercoralis.*
