# Supplementary material for: The epidemiology of soil-transmitted helminth infections in children up to 8 years of age: Findings from an Ecuadorian birth cohort
Source: PLoS Negl Trop Dis. 2021 Nov 19;15(11):e0009972. doi: 10.1371/journal.pntd.0009972 (PMC8641893; doi:10.1371/journal.pntd.0009972)
Supplement: S2 Table — Predicted data were derived from models fitted using population-averaged generalized estimating equations. (DOCX) [file pntd.0009972.s002.docx]

|  | **Any STH** | | | | ***A. lumbricoides*** | | | | ***T. trichiura*** | | | |
| --- | --- | --- | --- | --- | --- | --- | --- | --- | --- | --- | --- | --- |
| **AGE (MONTHS)** | **Raw** | **Prop** | **Low** | **High** | **Raw** | **Prop** | **Low** | **High** | **Raw prop** | **Estimated Prop** | **Low** | **High** |
| 7 | 0.020 | 0.025 | 0.019 | 0.031 | 0.018 | 0.020 | 0.014 | 0.025 | 0.003 | 0.008 | 0.004 | 0.011 |
| 13 | 0.126 | 0.106 | 0.089 | 0.122 | 0.097 | 0.080 | 0.066 | 0.094 | 0.035 | 0.031 | 0.022 | 0.041 |
| 18 | 0.196 | 0.186 | 0.160 | 0.212 | 0.138 | 0.138 | 0.116 | 0.160 | 0.085 | 0.062 | 0.047 | 0.077 |
| 24 | 0.216 | 0.235 | 0.208 | 0.263 | 0.142 | 0.169 | 0.146 | 0.193 | 0.117 | 0.095 | 0.078 | 0.112 |
| 30 | 0.235 | 0.232 | 0.205 | 0.258 | 0.148 | 0.161 | 0.137 | 0.184 | 0.131 | 0.111 | 0.094 | 0.128 |
| 36 | 0.246 | 0.206 | 0.178 | 0.234 | **0.162** | 0.138 | 0.113 | 0.164 | 0.145 | 0.115 | 0.094 | 0.135 |
| 60 | 0.251 | 0.258 | 0.224 | 0.292 | 0.153 | 0.178 | 0.146 | 0.209 | **0.165** | 0.149 | 0.122 | 0.176 |
| 96 | 0.204 | 0.183 | 0.154 | 0.212 | 0.122 | 0.105 | 0.083 | 0.128 | 0.133 | 0.106 | 0.086 | 0.126 |

S2 Table. Raw data and predicted age-dependent risk of infections with soil-transmitted helminths in ECUAVIDA. Predicted data were derived from models fitted using population-averaged generalized estimating equations.
