# Supplementary material for: The epidemiology of soil-transmitted helminth infections in children up to 8 years of age: Findings from an Ecuadorian birth cohort
Source: PLoS Negl Trop Dis. 2021 Nov 19;15(11):e0009972. doi: 10.1371/journal.pntd.0009972 (PMC8641893; doi:10.1371/journal.pntd.0009972)
Supplement: S3 Table — Estimates show population-averaged estimates using generalized estimating equations. *Time-varying variables. ‡Anthelmintic treatment during the previous 12 months. N-Afro.–non-Afro-Ecuadorian; Prim.–primary completed; Second.–secondary completed; SES–socioeconomic status; overcrowding–persons/sleeping room; Income- monthly household income; Non-trad.–non-traditional (wall construction with cement/blocks); trad.–traditional (wall construction with wood/bamboo); material goods–number of household electrical goods; pigs–keeping pigs around the house; agriculture–child lives on a farm or visits a farm at least once a week; NEG- negative; MOD-HEAVY–moderate and heavy intensity infections. (DOCX) [file pntd.0009972.s003.docx]

|  | ***A. lumbricoides*** | | **AGE-ADJUSTED** | | | | **MULTIVARIABLE** | | | |
| --- | --- | --- | --- | --- | --- | --- | --- | --- | --- | --- |
|  | **VARIABLE** | **CATEGORY** | **OR** | **p-value** | **95%CI**  **LOW** | **95%CI**  **HIGH** | **OR** | **p-value** | **95%CI**  **LOW** | **95%CI**  **HIGH** |
| **CHILDHOOD FACTORS** | **AGE** | **EFFECT OF 1 MONTH** | **3.587** | **<0.001** | **2.441** | **5.271** | **3.234** | **<0.001** | **2.130** | **4.910** |
|  | **AGE^2^** | **(NONLINEAR)** | **0.931** | **<0.001** | **0.907** | **0.957** | **0.938** | **<0.001** | **0.910** | **0.966** |
|  | **AGE^3^** |  | **1.002** | **<0.001** | **1.001** | **1.003** | **1.002** | **0.001** | **1.001** | **1.003** |
|  |  |  |  |  |  |  |  |  |  |  |
|  | **GENDER** | **Female vs. Male** | 0.923 | 0.519 | 0.725 | 1.176 |  |  |  |  |
|  | **BIRTH ORDER** | **3^rd^ -4^th^ vs. 1^st^ -2^nd^** | **1.378** | **0.011** | **1.078** | **1.763** |  |  |  |  |
|  |  | **>=5^th^ vs.1^st^ -2^nd^** | **2.263** | **<0.001** | **1.648** | **3.107** |  |  |  |  |
|  | **BREAST FEEDING (months)** | **7-12 vs.0-6** | 0.819 | 0.35 | 0.538 | 1.246 |  |  |  |  |
|  |  | **>12 vs.0-6** | 0.721 | 0.122 | 0.476 | 1.092 |  |  |  |  |
|  | **DAY CARE 36M** | **Yes vs. No** | **1.264** | **0.048** | **1.002** | **1.596** |  |  |  |  |
|  | ***RECENT TREATMENT** | **Yes vs. No** | **0.730** | **0.001** | **0.602** | **0.886** |  |  |  |  |
| **MATERNAL FACTORS** | **AGE (years)** | **21-29 vs. <=20** | 1.057 | 0.706 | 0.792 | 1.412 |  |  |  |  |
|  |  | **>=30 vs. <=20** | 0.868 | 0.399 | 0.626 | 1.205 |  |  |  |  |
|  | **ETHNICITY** | **NON-AFRO. vs. AFRO.** | **0.546** | **<0.001** | **0.433** | **0.689** | **0.615** | **<0.001** | **0.475** | **0.797** |
|  | **EDUCATION** | **PRIMARY vs. ILLITERATE** | **0.465** | **<0.001** | **0.348** | **0.622** | **0.487** | **<0.001** | **0.358** | **0.663** |
|  |  | **SECONDARY vs. ILLITERATE** | **0.218** | **<0.001** | **0.157** | **0.303** | **0.296** | **<0.001** | **0.204** | **0.431** |
|  | **ALLERGIC SYMPTOMS** | **Yes vs. No** | 0.863 | 0.531 | 0.544 | 1.369 |  |  |  |  |
|  | **ATOPY** | **Yes vs. No** | 1.007 | 0.958 | 0.762 | 1.332 |  |  |  |  |
| **PATERNAL FACTORS** | **AGE (years)** | **21-29 vs. <=20** | 0.771 | 0.271 | 0.486 | 1.225 |  |  |  |  |
|  |  | **>=30 vs. <=20** | 0.847 | 0.486 | 0.530 | 1.352 |  |  |  |  |
|  | **ETHNICITY** | **NON-AFRO. vs. AFRO.** | **0.632** | **<0.001** | **0.499** | **0.801** |  |  |  |  |
|  | **EDUCATION** | **PRIMARY vs. ILLITERATE** | **0.570** | **0.001** | **0.411** | **0.792** |  |  |  |  |
|  |  | **SECONDARY vs. ILLITERATE** | **0.423** | **<0.001** | **0.283** | **0.631** |  |  |  |  |
|  | **ALLERGIC SYMPTOMS** | **Yes vs. No** | 0.473 | **0.036** | 0.235 | 0.951 |  |  |  |  |
|  | **ATOPY** | **Yes vs. No** | 1.118 | 0.514 | 0.800 | 1.562 |  |  |  |  |
| **HOUSEHOLD SOCIO-ECONOMIC FACTORS** | **AREA OF RESIDENCE** | **RURAL vs. URBAN** | 1.117 | 0.386 | 0.869 | 1.436 |  |  |  |  |
|  | **SES** | **MED vs. LOW** | 0.800 | 0.11 | 0.608 | 1.052 |  |  |  |  |
|  |  | **HIGH vs. LOW** | **0.475** | **<0.001** | **0.351** | **0.643** |  |  |  |  |
|  | **OVERCROWDING** | **>=3 vs. <3** | **1.932** | **<0.001** | **1.525** | **2.449** | **1.448** | **0.006** | **1.111** | **1.888** |
|  | **INCOME** | **>1 vs. <1** | **0.861** | **0.006** | **0.773** | **0.958** |  |  |  |  |
|  | **HOUSE CONSTRUCTION** | **NON-TRAD. vs. TRAD.** | **0.671** | **0.002** | **0.520** | **0.866** |  |  |  |  |
|  | **MATERIAL GOODS** | **3-4 vs. 0-2** | **0.692** | **0.003** | **0.543** | **0.882** |  |  |  |  |
|  | **POTABLE WATER** | **Yes vs. No** | 1.082 | 0.518 | 0.852 | 1.374 |  |  |  |  |
|  | ***BATHROOM** | **Yes vs. No** | **0.672** | **<0.001** | **0.544** | **0.829** |  |  |  |  |
|  | **DOG IN HOUSE** | **Yes vs. No** | 0.951 | 0.756 | 0.695 | 1.303 |  |  |  |  |
|  | **CAT IN HOUSE** | **Yes vs. No** | 1.102 | 0.553 | 0.799 | 1.520 |  |  |  |  |
|  | ***PIGS** | **Yes vs. No** | 1.080 | 0.542 | 0.843 | 1.384 |  |  |  |  |
|  | **AGRICULTURAL EXPOSURE** | **Yes vs. No** | 0.817 | 0.099 | 0.643 | 1.039 |  |  |  |  |
| **HOUSEHOLD *A. lumbricoides*** | **MOTHER** | **Yes vs. No** | **2.664** | **<0.001** | **2.075** | **3.419** |  |  |  |  |
|  | **MOTHER INTENSITY** | **LIGHT vs. NEG** | **2.359** | **<0.001** | **1.713** | **3.250** |  |  |  |  |
|  |  | **MOD/HEAVY vs. NEG** | **6.071** | **<0.001** | **4.372** | **8.428** |  |  |  |  |
|  | **FATHER** | **Yes vs. No** | **2.019** | **0.002** | **1.303** | **3.128** |  |  |  |  |
|  | **ANY HOUSEHOLD** | **Yes vs. No** | **2.587** | **<0.001** | **2.017** | **3.317** | **2.041** | **<0.001** | **1.592** | **2.617** |
|  | **ANY EXCEPT PARENTS** | **Yes vs. No** | **2.486** | **<0.001** | **1.844** | **3.352** |  |  |  |  |
|  | **SIBLINGS** | **Yes vs. No** | **2.707** | **<0.001** | **1.937** | **3.782** |  |  |  |  |

S3 Table. Age-adjusted and multivariable associations between *A. lumbricoides* infection during first 8 years of life and individual, parental, and household factors including *A. lumbricoides* infections among household members. Estimates show population-averaged estimates using generalized estimating equations.

*Time-varying variables. ‡Anthelmintic treatment during the previous 12 months. N-Afro. – non-Afro-Ecuadorian; Prim. – primary completed; Second. – secondary completed; SES – socioeconomic status; overcrowding – persons/sleeping room; Income- monthly household income; Non-trad. – non-traditional (wall construction with cement/blocks); trad. – traditional (wall construction with wood/bamboo); material goods – number of household electrical goods; pigs – keeping pigs around the house; agriculture – child lives on a farm or visits a farm at least once a week; NEG- negative; MOD-HEAVY – moderate and heavy intensity infections.
