# Supplementary material for: The epidemiology of soil-transmitted helminth infections in children up to 8 years of age: Findings from an Ecuadorian birth cohort
Source: PLoS Negl Trop Dis. 2021 Nov 19;15(11):e0009972. doi: 10.1371/journal.pntd.0009972 (PMC8641893; doi:10.1371/journal.pntd.0009972)
Supplement: S4 Table — Estimates show population-averaged estimates using generalized estimating equations. *Time-varying variables. ‡Anthelmintic treatment during the previous 12 months. N-Afro.–non-Afro-Ecuadorian; Prim.–primary completed; Second.–secondary completed; SES–socioeconomic status; overcrowding–persons/sleeping room; Income- monthly household income; Non-trad.–non-traditional (wall construction with cement/blocks); trad.–traditional (wall construction with wood/bamboo); material goods–number of household electrical goods; pigs–keeping pigs around the house; agriculture–child lives on a farm or visits a farm at least once a week; NEG- negative; MOD-HEAVY–moderate and heavy intensity infections. (DOCX) [file pntd.0009972.s004.docx]

|  | ***T. trichiura*** | | **AGE-ADJUSTED** | | | | **MULTIVARIABLE** | | | |
| --- | --- | --- | --- | --- | --- | --- | --- | --- | --- | --- |
|  | **VARIABLE** | **CATEGORY** | **OR** | **p-value** | **95%CI**  **LOW** | **95%CI**  **HIGH** | **OR** | **p-value** | **95%CI**  **LOW** | **95%CI**  **HIGH** |
| **CHILDHOOD FACTORS** | **AGE** | **EFFECT OF 1 MONTH** | **7.901** | **<0.001** | **3.843** | **16.242** | **7.560** | **<0.001** | **2.996** | **19.270** |
|  | **AGE^2^** | **(NONLINEAR)** | **0.891** | **<0.001** | **0.852** | **0.932** | **0.890** | **<0.001** | **0.838** | **0.946** |
|  | **AGE^3^** |  | **1.003** | **<0.001** | **1.002** | **1.004** | **1.003** | **<0.001** | **1.001** | **1.005** |
|  |  |  |  |  |  |  |  |  |  |  |
|  | **GENDER** | **Female vs. Male** | 1.219 | 0.21 | 0.894 | 1.661 |  |  |  |  |
|  | **BIRTH ORDER** | **3^rd^ -4^th^ vs. 1^st^ -2^nd^** | **1.430** | **0.026** | **1.044** | **1.959** |  |  |  |  |
|  |  | **>=5^th^ vs.1^st^ -2^nd^** | **2.720** | **<0.001** | **1.774** | **4.169** |  |  |  |  |
|  | **BREAST FEEDING (months)** | **7-12 vs.0-6** | 0.975 | 0.916 | 0.609 | 1.562 |  |  |  |  |
|  |  | **>12 vs.0-6** | 0.833 | 0.397 | 0.546 | 1.271 |  |  |  |  |
|  | **DAY CARE 36M** | **Yes vs. No** | **1.574** | **0.007** | **1.134** | **2.184** |  |  |  |  |
|  | ***RECENT TREATMENT** | **Yes vs. No** | 0.847 | 0.268 | 0.631 | 1.137 |  |  |  |  |
| **MATERNAL FACTORS** | **AGE (years)** | **21-29 vs. <=20** | 1.287 | 0.163 | 0.903 | 1.834 |  |  |  |  |
|  |  | **>=30 vs. <=20** | 0.932 | 0.694 | 0.658 | 1.322 |  |  |  |  |
|  | **ETHNICITY** | **NON-AFRO. vs. AFRO.** | **0.437** | **<0.001** | **0.324** | **0.589** | **0.490** | **0.003** | **0.306** | **0.784** |
|  | **EDUCATION** | **PRIMARY vs. ILLITERATE** | **0.445** | **<0.001** | **0.294** | **0.673** | 0.619 | 0.083 | 0.359 | 1.065 |
|  |  | **SECONDARY vs. ILLITERATE** | **0.233** | **<0.001** | **0.141** | **0.385** | **0.470** | **0.021** | **0.248** | **0.891** |
|  | **ALLERGIC SYMPTOMS** | **Yes vs. No** | 0.807 | 0.533 | 0.411 | 1.583 |  |  |  |  |
|  | **ATOPY** | **Yes vs. No** | **0.682** | **0.02** | **0.494** | **0.942** |  |  |  |  |
| **PATERNAL FACTORS** | **AGE (years)** | **21-29 vs. <=20** | 0.900 | 0.686 | 0.539 | 1.502 |  |  |  |  |
|  |  | **>=30 vs. <=20** | 1.172 | 0.559 | 0.688 | 1.995 |  |  |  |  |
|  | **ETHNICITY** | **NON-AFRO. vs. AFRO.** | **0.431** | **<0.001** | **0.316** | **0.587** |  |  |  |  |
|  | **EDUCATION** | **PRIMARY vs. ILLITERATE** | **0.450** | **0.001** | **0.278** | **0.728** |  |  |  |  |
|  |  | **SECONDARY vs. ILLITERATE** | **0.292** | **<0.001** | **0.176** | **0.486** |  |  |  |  |
|  | **ALLERGIC SYMPTOMS** | **Yes vs. No** | 1.296 | 0.529 | 0.578 | 2.905 |  |  |  |  |
|  | **ATOPY** | **Yes vs. No** | 0.835 | 0.436 | 0.531 | 1.314 |  |  |  |  |
| **HOUSEHOLD SOCIO-ECONOMIC FACTORS** | **AREA OF RESIDENCE** | **RURAL vs. URBAN** | 0.812 | 0.249 | 0.571 | 1.156 |  |  |  |  |
|  | **SES** | **MED vs. LOW** | 0.700 | 0.068 | 0.477 | 1.027 | 0.752 | 0.186 | 0.493 | 1.147 |
|  |  | **HIGH vs. LOW** | **0.450** | **<0.001** | **0.314** | **0.645** | **0.631** | **0.047** | **0.400** | **0.994** |
|  | **OVERCROWDING** | **>=3 vs. <3** | **2.655** | **<0.001** | **1.955** | **3.607** | **1.770** | **0.009** | **1.155** | **2.711** |
|  | **INCOME** | **>1 vs. <1** | 0.865 | 0.087 | 0.733 | 1.021 |  |  |  |  |
|  | **HOUSE CONSTRUCTION** | **NON-TRAD. vs. TRAD.** | **0.586** | **0.008** | **0.394** | **0.871** |  |  |  |  |
|  | **MATERIAL GOODS** | **3-4 vs. 0-2** | **0.550** | **<0.001** | **0.413** | **0.733** |  |  |  |  |
|  | **POTABLE WATER** | **Yes vs. No** | 1.107 | 0.507 | 0.820 | 1.495 |  |  |  |  |
|  | ***BATHROOM** | **Yes vs. No** | 0.866 | 0.279 | 0.667 | 1.124 |  |  |  |  |
|  | **DOG IN HOUSE** | **Yes vs. No** | 1.160 | 0.403 | 0.820 | 1.641 |  |  |  |  |
|  | **CAT IN HOUSE** | **Yes vs. No** | 1.121 | 0.658 | 0.675 | 1.861 |  |  |  |  |
|  | ***PIGS** | **Yes vs. No** | 1.135 | 0.542 | 0.755 | 1.708 |  |  |  |  |
|  | **AGRICULTURAL EXPOSURE** | **Yes vs. No** | 0.911 | 0.559 | 0.667 | 1.245 |  |  |  |  |
| **HOUSEHOLD *T. trichiura*** | **MOTHER** | **Yes vs. No** | **3.861** | **<0.001** | **3.220** | **4.629** |  |  |  |  |
|  | **MOTHER INTENSITY** | **LIGHT vs. NEG** | **2.410** | **<0.001** | **1.683** | **3.452** |  |  |  |  |
|  |  | **MOD/HEAVY vs. NEG** | **18.257** | **<0.001** | **9.142** | **36.459** |  |  |  |  |
|  | **FATHER** | **Yes vs. No** | **2.827** | **<0.001** | **1.610** | **4.966** |  |  |  |  |
|  | **ANY HOUSEHOLD** | **Yes vs. No** | **3.569** | **<0.001** | **2.587** | **4.924** |  |  |  |  |
|  | **ANY EXCEPT PARENTS** | **Yes vs. No** | **4.422** | **<0.001** | **2.965** | **6.595** |  |  |  |  |
|  | **SIBLINGS** | **Yes vs. No** | **5.092** | **<0.001** | **3.229** | **8.028** | **3.415** | **<0.001** | **2.236** | **5.215** |

S4 Table. Age-adjusted and multivariable associations between *T. trichiura* infections during first 8 years of life and individual, parental, and household factors including *T. trichiura* infections among household members. Estimates show population-averaged estimates using generalized estimating equations.

*Time-varying variables. ‡Anthelmintic treatment during the previous 12 months. N-Afro. – non-Afro-Ecuadorian; Prim. – primary completed; Second. – secondary completed; SES – socioeconomic status; overcrowding – persons/sleeping room; Income- monthly household income; Non-trad. – non-traditional (wall construction with cement/blocks); trad. – traditional (wall construction with wood/bamboo); material goods – number of household electrical goods; pigs – keeping pigs around the house; agriculture – child lives on a farm or visits a farm at least once a week; NEG- negative; MOD-HEAVY – moderate and heavy intensity infections.
