# Supplementary material for: The epidemiology of soil-transmitted helminth infections in children up to 8 years of age: Findings from an Ecuadorian birth cohort
Source: PLoS Negl Trop Dis. 2021 Nov 19;15(11):e0009972. doi: 10.1371/journal.pntd.0009972 (PMC8641893; doi:10.1371/journal.pntd.0009972)
Supplement: S5 Table — A. lumbricoides infection intensity was measured as eggs per gramme (epg) of stool. Original epg counts were fit using a zero-inflated model. Zeros model represents associations of a positive count with variables (denoted by 1 in our previous analyses) (OR>1 indicates a positive association with positive counts while <1 indicates an association with zero counts). Counts model represents associations using the negative binomial distribution (Fold-change>1 indicates fold increase in egg counts associated with that variable while <1 indicates corresponding fold decrease). (DOCX) [file pntd.0009972.s005.docx]

|  | ***A. lumbricoides*** | | **ZEROS MODEL** | | | | **COUNTS MODEL** | | | |
| --- | --- | --- | --- | --- | --- | --- | --- | --- | --- | --- |
|  | **VARIABLE** | **CATEGORY** | **OR** | **p-value** | **95%CI**  **LOW** | **95%CI**  **HIGH** | **Fold-change** | **p-value** | **95%CI**  **LOW** | **95%CI**  **HIGH** |
| **CHILDHOOD FACTORS** | **AGE** | **EFFECT OF 1 MONTH** | **2.976** | **<0.001** | **1.551** | **4.401** | 1.019 | 0.173 | 0.992 | 1.047 |
|  | **AGE^2^** | **(NONLINEAR)** | **0.943** | **<0.001** | **0.991** | **0.974** | 1.000 | 0.106 | 1.000 | 1.000 |
|  | **AGE^3^** |  | **1.001** | **0.005** | **1.000** | **1.003** |  |  |  |  |
|  |  |  |  |  |  |  |  |  |  |  |
|  | **GENDER** | **Female vs. Male** | 0.894 | 0.402 | 0.659 | 1.128 | **1.587** | **0.008** | **1.128** | **2.232** |
|  | **BIRTH ORDER** | **3^rd^ -4^th^ vs. 1^st^ -2^nd^** | **1.313** | **0.038** | **0.976** | **1.650** | 1.010 | 0.961 | 0.671 | 1.520 |
|  |  | **>=5^th^ vs. 1^st^ -2^nd^** | **2.269** | **<0.001** | **1.508** | **3.031** | **1.589** | **0.031** | **1.042** | **2.422** |
|  | **BREAST FEEDING (months)** | **7-12 vs.0-6** | 0.812 | 0.351 | 0.457 | 1.167 | 1.346 | 0.342 | 0.729 | 2.484 |
|  |  | **>12 vs.0-6** | 0.710 | 0.124 | 0.401 | 1.020 | 1.224 | 0.516 | 0.665 | 2.250 |
|  | **DAY CARE 36M** | **Yes vs. No** | **1.280** | **0.045** | **0.970** | **1.590** | 1.318 | 0.151 | 0.904 | 1.920 |
|  | ***RECENT TREATMENT** | **Yes vs. No** | **0.719** | **0.012** | **0.534** | **0.904** | 1.356 | 0.145 | 0.900 | 2.041 |
| **MATERNAL FACTORS** | **AGE (years)** | **21-29 vs. <=20** | 1.070 | 0.648 | 0.759 | 1.381 | 1.123 | 0.566 | 0.755 | 1.671 |
|  |  | **>=30 vs. <=20** | 0.869 | 0.427 | 0.568 | 1.170 | **1.623** | **0.025** | **1.062** | **2.479** |
|  | **ETHNICITY** | **NON-AFRO. vs. AFRO.** | **0.493** | **<0.001** | **0.414** | **0.572** | **0.621** | **0.003** | **0.455** | **0.849** |
|  | **EDUCATION** | **PRIMARY vs. ILLITERATE** | **0.482** | **<0.001** | **0.393** | **0.571** | **0.626** | **0.003** | **0.457** | **0.856** |
|  |  | **SECONDARY vs. ILLITERATE** | **0.198** | **<0.001** | **0.149** | **0.248** | 0.687 | 0.310 | 0.332 | 1.420 |
|  | **ALLERGIC SYMPTOMS** | **Yes vs. No** | 0.980 | 0.914 | 0.623 | 1.338 | 1.493 | 0.228 | 0.779 | 2.863 |
|  | **ATOPY** | **Yes vs. No** | 1.001 | 0.991 | 0.815 | 1.187 | 0.890 | 0.506 | 0.632 | 1.254 |
| **PATERNAL FACTORS** | **AGE (years)** | **21-29 vs. <=20** | 0.730 | 0.170 | 0.401 | 1.058 | 1.205 | 0.526 | 0.677 | 2.143 |
|  |  | **>=30 vs. <=20** | 0.802 | 0.347 | 0.434 | 1.170 | 1.575 | 0.113 | 0.898 | 2.764 |
|  | **ETHNICITY** | **NON-AFRO. vs. AFRO.** | **0.612** | **<0.001** | **0.506** | **0.718** | 0.735 | 0.077 | 0.522 | 1.034 |
|  | **EDUCATION** | **PRIMARY vs. ILLITERATE** | **0.617** | **<0.001** | **0.492** | **0.743** | **0.638** | **0.016** | **0.442** | **0.921** |
|  |  | **SECONDARY vs. ILLITERATE** | **0.352** | **<0.001** | **0.264** | **0.439** | **0.547** | **0.004** | **0.362** | **0.824** |
|  | **ALLERGIC SYMPTOMS** | **Yes vs. No** | **0.521** | **0.017** | **0.242** | **0.800** | 0.704 | 0.381 | 0.320 | 1.546 |
|  | **ATOPY** | **Yes vs. No** | 0.888 | 0.324 | 0.678 | 1.098 | 1.443 | 0.222 | 0.801 | 2.597 |
| **HOUSEHOLD SOCIO-ECONOMIC FACTORS** | **AREA OF RESIDENCE** | **RURAL vs. URBAN** | 1.175 | 0.233 | 0.864 | 1.486 | 0.786 | 0.215 | 0.537 | 1.150 |
|  | **SES** | **MED vs. LOW** | 0.844 | 0.272 | 0.589 | 1.099 | 0.666 | 0.029 | 0.462 | 0.960 |
|  |  | **HIGH vs. LOW** | **0.464** | **<0.001** | **0.321** | **0.607** | 0.603 | 0.040 | 0.372 | 0.978 |
|  | **OVERCROWDING** | **>=3 vs. <3** | **2.025** | **<0.001** | **1.507** | **2.543** | 1.274 | 0.206 | 0.875 | 1.855 |
|  | **INCOME** | **>1 vs. <1** | **0.829** | **0.001** | **0.736** | **0.922** | 0.974 | 0.787 | 0.804 | 1.180 |
|  | **HOUSE CONSTRUCTION** | **NON-TRAD. vs. TRAD.** | **0.719** | **<0.001** | **0.526** | **0.912** | 0.784 | 0.190 | 0.545 | 1.128 |
|  | **MATERIAL GOODS** | **3-4 vs. 0-2** | **0.708** | **0.009** | **0.525** | **0.891** | **0.691** | **0.049** | **0.478** | **0.999** |
|  | **POTABLE WATER** | **Yes vs. No** | 1.037 | 0.769 | 0.783 | 1.292 | 0.872 | 0.451 | 0.610 | 1.246 |
|  | ***BATHROOM** | **Yes vs. No** | **0.536** | **<0.001** | **0.272** | **0.799** | **0.650** | **0.016** | **0.457** | **0.923** |
|  | **DOG IN HOUSE** | **Yes vs. No** | 0.886 | 0.481 | 0.587 | 1.185 | 1.164 | 0.547 | 0.710 | 1.906 |
|  | **CAT IN HOUSE** | **Yes vs. No** | 1.052 | 0.756 | 0.715 | 1.390 | 1.247 | 0.413 | 0.735 | 2.116 |
|  | ***PIGS** | **Yes vs. No** | 1.178 | 0.162 | 0.907 | 1.449 | **1.488** | **0.031** | **1.037** | **2.137** |
|  | **AGRICULTURAL EXPOSURE** | **Yes vs. No** | 0.864 | 0.256 | 0.645 | 1.082 | 0.910 | 0.613 | 0.632 | 1.311 |
| **HOUSEHOLD *A. lumbricoides*** | **MOTHER** | **Yes vs. No** | **2.859** | **<0.001** | **2.040** | **3.679** | **1.815** | **0.008** | **1.172** | **2.810** |
|  | **MOTHER INTENSITY** | **LIGHT vs. NEG** | **2.361** | **<0.001** | **1.549** | **3.172** | **1.544** | **0.028** | **1.048** | **2.273** |
|  |  | **MOD/HEAVY vs. NEG** | **6.182** | **<0.001** | **4.240** | **8.124** | **2.698** | **<0.001** | **1.730** | **4.207** |
|  | **FATHER** | **Yes vs. No** | **2.085** | **0.003** | **1.068** | **3.101** | 1.914 | 0.067 | 0.954 | 3.838 |
|  | **ANY HOUSEHOLD** | **Yes vs. No** | **2.627** | **<0.001** | **1.938** | **3.315** | **1.973** | **0.002** | **1.272** | **3.061** |
|  | **ANY EXCEPT PARENTS** | **Yes vs. No** | **2.642** | **<0.001** | **1.870** | **3.415** | 1.612 | 0.060 | 0.980 | 2.652 |
|  | **SIBLINGS** | **Yes vs. No** | **2.871** | **<0.001** | **1.922** | **3.821** | **1.893** | **0.016** | **1.127** | **3.182** |

S5 Table. Age-adjusted associations between *A. lumbricoides* infection intensity during first 8 years of life and individual, parental, and household factors including *A. lumbricoides* infections among household members. *A. lumbricoides* infection intensity was measured as eggs per gramme (epg) of stool. Original epg counts were fit using a zero-inflated model. Zeros model represents associations of a positive count with variables (denoted by 1 in our previous analyses) (OR>1 indicates a positive association with positive counts while <1 indicates an association with zero counts). Counts model represents associations using the negative binomial distribution (Fold-change>1 indicates fold increase in egg counts associated with that variable while <1 indicates corresponding fold decrease).
