# Supplementary material for: The epidemiology of soil-transmitted helminth infections in children up to 8 years of age: Findings from an Ecuadorian birth cohort
Source: PLoS Negl Trop Dis. 2021 Nov 19;15(11):e0009972. doi: 10.1371/journal.pntd.0009972 (PMC8641893; doi:10.1371/journal.pntd.0009972)
Supplement: S6 Table — T. trichiura infection intensity was measured as eggs per gramme (epg) of stool. Original epg counts were fit using a zero-inflated model. Zeros model represents associations of a positive count with variables (denoted by 1 in our previous analyses) (OR>1 indicates a positive association with positive counts while <1 indicates an association with zero counts). Counts model represents associations using the negative binomial distribution (Fold-change>1 indicates fold increase in egg counts associated with that variable while <1 indicates corresponding fold decrease). (DOCX) [file pntd.0009972.s006.docx]

|  | ***T. trichiura*** | | **ZEROS MODEL** | | | | **COUNTS MODEL** | | | |
| --- | --- | --- | --- | --- | --- | --- | --- | --- | --- | --- |
|  | **VARIABLE** | **CATEGORY** | **OR** | **p-value** | **95%CI**  **LOW** | **95%CI**  **HIGH** | **Fold-change** | **p-value** | **95%CI**  **LOW** | **95%CI**  **HIGH** |
| **CHILDHOOD FACTORS** | **AGE** | **EFFECT OF 1 MONTH** | **6.527** | **0.009** | **1.663** | **11.390** | 1.052 | 0.160 | 0.980 | 1.128 |
|  | **AGE^2^** | **(NONLINEAR)** | **0.904** | **<0.001** | **0.861** | **0.946** | 1.000 | 0.187 | 0.999 | 1.000 |
|  | **AGE^3^** |  | **1.003** | **<0.001** | **1.001** | **1.004** |  |  |  |  |
|  |  |  |  |  |  |  |  |  |  |  |
|  | **GENDER** | **Female vs. Male** | 1.377 | 0.107 | 0.841 | 1.913 | **2.193** | **0.044** | **1.021** | **4.709** |
|  | **BIRTH ORDER** | **3^rd^ -4^th^ vs. 1^st^ -2^nd^** | 1.405 | 0.033 | 0.966 | 1.845 | 0.784 | 0.543 | 0.358 | 1.718 |
|  |  | **>=5^th^ vs.1^st^ -2^nd^** | **3.190** | **<0.001** | **1.555** | **4.826** | **2.965** | **0.024** | **1.152** | **7.633** |
|  | **BREAST FEEDING (months)** | **7-12 vs.0-6** | 1.020 | 0.945 | 0.454 | **1.586** | **4.630** | **<0.001** | **2.266** | **9.458** |
|  |  | **>12 vs.0-6** | 0.760 | 0.223 | 0.424 | **1.096** | **1.905** | **0.025** | **1.086** | **3.339** |
|  | **DAY CARE 36M** | **Yes vs. No** | 1.434 | 0.054 | 0.908 | 1.961 | 0.731 | 0.441 | 0.330 | 1.621 |
|  | ***RECENT TREATMENT** | **Yes vs. No** | 0.962 | 0.792 | 0.687 | 1.238 | **0.404** | **0.011** | **0.202** | **0.810** |
| **MATERNAL FACTORS** | **AGE (years)** | **21-29 vs. <=20** | 1.504 | 0.047 | 0.898 | 2.110 | **3.889** | **0.001** | **1.757** | **8.610** |
|  |  | **>=30 vs. <=20** | 0.974 | 0.881 | 0.643 | 1.306 | **2.506** | **0.006** | **1.295** | **4.848** |
|  | **ETHNICITY** | **NON-AFRO. vs. AFRO.** | **0.476** | **<0.001** | **0.381** | **0.570** | 0.898 | 0.745 | 0.469 | 1.718 |
|  | **EDUCATION** | **PRIMARY vs. ILLITERATE** | **0.484** | **<0.001** | **0.371** | **0.598** | 0.600 | 0.141 | 0.304 | 1.184 |
|  |  | **SECONDARY vs. ILLITERATE** | **0.220** | **<0.001** | **0.153** | **0.288** | **0.365** | **0.002** | **0.192** | **0.692** |
|  | **ALLERGIC SYMPTOMS** | **Yes vs. No** | 0.801 | 0.406 | 0.381 | 1.220 | 0.616 | 0.203 | 0.292 | 1.299 |
|  | **ATOPY** | **Yes vs. No** | 1.014 | 0.902 | 0.787 | 1.242 | 0.638 | 0.083 | 0.384 | 1.061 |
| **PATERNAL FACTORS** | **AGE (years)** | **21-29 vs. <=20** | 0.482 | 0.715 | 0.383 | 0.582 | 0.764 | 0.433 | 0.391 | 1.496 |
|  |  | **>=30 vs. <=20** | 0.917 | 0.294 | 0.492 | 1.342 | 1.415 | 0.173 | 0.859 | 2.329 |
|  | **ETHNICITY** | **NON-AFRO. vs. AFRO.** | **1.331** | **<0.001** | **0.621** | **2.042** | **4.187** | **<0.001** | **2.005** | **8.745** |
|  | **EDUCATION** | **PRIMARY vs. ILLITERATE** | **0.494** | **<0.001** | **0.368** | **0.621** | 0.755 | 0.371 | 0.408 | 1.397 |
|  |  | **SECONDARY vs. ILLITERATE** | **0.310** | **<0.001** | **0.216** | **0.403** | **0.488** | **0.032** | **0.253** | **0.938** |
|  | **ALLERGIC SYMPTOMS** | **Yes vs. No** | 0.902 | 0.727 | 0.380 | 1.424 | 0.792 | 0.607 | 0.326 | 1.924 |
|  | **ATOPY** | **Yes vs. No** | 0.779 | 0.087 | 0.555 | 1.002 | 1.160 | 0.598 | 0.669 | 2.010 |
| **HOUSEHOLD SOCIO-ECONOMIC FACTORS** | **AREA OF RESIDENCE** | **RURAL vs. URBAN** | **0.822** | **<0.001** | **0.419** | **1.225** | **2.515** | **0.021** | **1.150** | **5.496** |
|  | **SES** | **MED vs. LOW** | 0.790 | 0.380 | 0.373 | 1.206 | 0.510 | 0.111 | 0.223 | 1.168 |
|  |  | **HIGH vs. LOW** | **0.448** | **<0.001** | **0.284** | **0.611** | **0.278** | **0.001** | **0.135** | **0.572** |
|  | **OVERCROWDING** | **>=3 vs. <3** | **2.984** | **<0.001** | **1.931** | **4.037** | **3.714** | **<0.001** | **2.023** | **6.819** |
|  | **INCOME** | **>1 vs. <1** | 0.861 | 0.183 | 0.671 | 1.051 | 1.367 | 0.174 | 0.871 | 2.144 |
|  | **HOUSE CONSTRUCTION** | **NON-TRAD. vs. TRAD.** | **0.596** | **0.016** | **0.346** | **0.846** | **0.366** | **0.013** | **0.165** | **0.811** |
|  | **MATERIAL GOODS** | **3-4 vs. 0-2** | **0.535** | **<0.001** | **0.351** | **0.718** | 0.497 | 0.132 | 0.200 | 1.233 |
|  | **POTABLE WATER** | **Yes vs. No** | **1.067** | **<0.001** | **0.700** | **1.435** | **0.460** | **0.025** | **0.233** | **0.907** |
|  | ***BATHROOM** | **Yes vs. No** | **0.566** | **<0.001** | **0.405** | **0.726** | 0.649 | 0.085 | 0.397 | 1.062 |
|  | **DOG IN HOUSE** | **Yes vs. No** | 1.185 | 0.392 | 0.724 | 1.645 | **0.369** | **0.002** | **0.197** | **0.690** |
|  | **CAT IN HOUSE** | **Yes vs. No** | 1.162 | 0.488 | 0.669 | 1.654 | **2.583** | **0.047** | **1.011** | **6.600** |
|  | ***PIGS** | **Yes vs. No** | 0.887 | 0.560 | 0.531 | 1.244 | 0.703 | 0.473 | 0.268 | 1.844 |
|  | **AGRICULTURAL EXPOSURE** | **Yes vs. No** | **0.822** | **<0.001** | **0.419** | **1.225** | **2.515** | **0.021** | **1.150** | **5.496** |
| **HOUSEHOLD *T. trichiura*** | **MOTHER** | **Yes vs. No** | **3.937** | **<0.001** | **2.267** | **5.606** | **6.597** | **<0.001** | **3.505** | **12.418** |
|  | **MOTHER INTENSITY** | **LIGHT vs. NEG** | **2.250** | **<0.001** | **1.384** | **3.115** | 1.002 | 0.995 | 0.514 | 1.954 |
|  |  | **MOD/HEAVY vs. NEG** | **16.616** | **0.008** | **4.436** | **28.796** | **7.579** | **<0.001** | **3.746** | **15.335** |
|  | **FATHER** | **Yes vs. No** | **2.678** | **<0.001** | **1.171** | **4.184** | 1.158 | 0.817 | 0.335 | 4.005 |
|  | **ANY HOUSEHOLD** | **Yes vs. No** | **3.499** | **<0.001** | **2.302** | **4.696** | 2.852 | 0.053 | 0.987 | 8.239 |
|  | **ANY EXCEPT PARENTS** | **Yes vs. No** | **4.212** | **<0.001** | **2.462** | **5.961** | **5.262** | **<0.001** | **2.594** | **10.673** |
|  | **SIBLINGS** | **Yes vs. No** | **4.854** | **<0.001** | **2.534** | **7.175** | **5.544** | **<0.001** | **2.674** | **11.493** |

S6 Table. Age-adjusted associations between *T. trichiura* infection intensity during first 8 years of life and individual, parental, and household factors including *T. trichiura* infections among household members. *T. trichiura* infection intensity was measured as eggs per gramme (epg) of stool. Original epg counts were fit using a zero-inflated model. Zeros model represents associations of a positive count with variables (denoted by 1 in our previous analyses) (OR>1 indicates a positive association with positive counts while <1 indicates an association with zero counts). Counts model represents associations using the negative binomial distribution (Fold-change>1 indicates fold increase in egg counts associated with that variable while <1 indicates corresponding fold decrease).
