# Supplementary material for: The epidemiology of soil-transmitted helminth infections in children up to 8 years of age: Findings from an Ecuadorian birth cohort
Source: PLoS Negl Trop Dis. 2021 Nov 19;15(11):e0009972. doi: 10.1371/journal.pntd.0009972 (PMC8641893; doi:10.1371/journal.pntd.0009972)
Supplement: S7 Table — GEE provide population average estimates while MLE provide subject-specific estimates. (DOCX) [file pntd.0009972.s007.docx]

| **ANY CHILDHOOD STH** | | **GEE ESTIMATES** | | | | **MLE ESTIMATES** | | | | |
| --- | --- | --- | --- | --- | --- | --- | --- | --- | --- | --- |
| **VARIABLE** | **CATEGORY** | **OR** | **p-value** | **95%CI**  **LOW** | **95%CI**  **HIGH** | | **OR** | **p-value** | **95%CI**  **LOW** | **95%CI**  **HIGH** |
| **AGE** | **EFFECT OF** | 4.073 | **<0.001** | 2.837 | 5.847 | | 5.357 | **<0.001** | 3.068 | 9.353 |
| **AGE^2^** | **1 MONTH** | 0.925 | **<0.001** | 0.903 | 0.948 | | 0.913 | **<0.001** | 0.880 | 0.947 |
| **AGE^3^** | **(NONLINEAR)** | 1.002 | **<0.001** | 1.001 | 1.003 | | 1.002 | **<0.001** | 1.001 | 1.003 |
| **SEX** | **F vs. M** | 1.038 | 0.753 | 0.825 | 1.306 | | 0.992 | 0.935 | 0.819 | 1.202 |
| **BIRTH ORDER** | **3-4 vs. 1-2** | 1.396 | **0.003** | 1.119 | 1.741 | | 1.516 | **<0.001** | 1.223 | 1.880 |
|  | **>=5 vs.1-2** | 2.461 | **<0.001** | 1.800 | 3.364 | | 3.069 | **<0.001** | 2.376 | 3.965 |
| **BREAST FEEDING** | **7-12 vs.0-6** | 0.904 | 0.606 | 0.615 | 1.328 | | 1.178 | 0.353 | 0.834 | 1.663 |
|  | **>12 vs.0-6** | 0.803 | 0.234 | 0.559 | 1.153 | | 1.043 | 0.810 | 0.742 | 1.464 |
| **DAY CARE 36M** | **Y vs. N** | 1.373 | **0.007** | 1.089 | 1.732 | | 2.027 | **<0.001** | 1.609 | 2.555 |
| **ANTIPATHERAPY** | **Y vs. N (time-vary)** | 0.782 | **0.012** | 0.647 | 0.947 | | 0.718 | **0.015** | 0.550 | 0.937 |
| **MATERNAL AGE** | **21-29 vs. <=20** | 1.156 | 0.284 | 0.887 | 1.507 | | 1.037 | 0.756 | 0.825 | 1.303 |
|  | **>=30 vs. <=20** | 0.924 | 0.571 | 0.705 | 1.213 | | 0.913 | 0.500 | 0.700 | 1.190 |
| **MATERNAL ETHNICITY** | **N-AFRO vs. AFRO** | 0.512 | **<0.001** | 0.410 | 0.639 | | 0.364 | **<0.001** | 0.298 | 0.446 |
| **MATERNAL EDU** | **PRIM vs. ILLIT** | 0.462 | **<0.001** | 0.345 | 0.619 | | 0.370 | **<0.001** | 0.285 | 0.479 |
|  | **SECOND vs. ILLIT** | 0.229 | **<0.001** | 0.167 | 0.314 | | 0.141 | **<0.001** | 0.103 | 0.192 |
| **MATERNAL ALLERGY** | **Y vs. N** | 0.706 | 0.131 | 0.449 | 1.110 | | 0.699 | 0.160 | 0.424 | 1.152 |
| **MATERNAL ATOPY** | **Y vs. N** | 0.908 | 0.471 | 0.698 | 1.181 | | 0.939 | 0.598 | 0.745 | 1.185 |
| **PATERNAL AGE** | **21-29 vs. <=20** | 0.835 | 0.33 | 0.581 | 1.200 | | 0.777 | 0.154 | 0.550 | 1.099 |
|  | **>=30 vs. <=20** | 0.964 | 0.847 | 0.661 | 1.405 | | 0.882 | 0.473 | 0.625 | 1.243 |
| **PATERNAL ETHNICITY** | **N-AFRO vs. AFRO** | 0.567 | **<0.001** | 0.450 | 0.714 | | 0.448 | **<0.001** | 0.361 | 0.557 |
| **PATERNAL EDUCATION** | **PRIM vs. ILLIT** | 0.517 | **<0.001** | 0.366 | 0.731 | | 0.479 | **<0.001** | 0.362 | 0.633 |
|  | **SECOND vs. ILLIT** | 0.364 | **<0.001** | 0.248 | 0.535 | | 0.261 | **<0.001** | 0.191 | 0.357 |
| **PATERNAL ALLERGY** | **Y vs. N** | 0.670 | 0.205 | 0.360 | 1.246 | | 0.601 | 0.113 | 0.320 | 1.129 |
| **PATERNAL ATOPY** | **Y vs. N** | 1.054 | 0.749 | 0.763 | 1.457 | | 0.835 | 0.205 | 0.632 | 1.103 |
| **ADMIN RESIDENCE** | **RURAL vs. URBAN** | 0.996 | 0.974 | 0.778 | 1.276 | | 0.783 | 0.028 | 0.630 | 0.974 |
| **SOCIO ECON STATUS** | **MED vs. LOW** | 0.750 | **0.04** | 0.570 | 0.988 | | 0.663 | **<0.001** | 0.528 | 0.832 |
|  | **HIGH vs. LOW** | 0.463 | **<0.001** | 0.356 | 0.602 | | 0.353 | **<0.001** | 0.278 | 0.448 |
| **HOUSE CROWDING** | **>=3 vs. <3** | 2.096 | **<0.001** | 1.676 | 2.622 | | 2.698 | **<0.001** | 2.227 | 3.268 |
| **MATERNAL INCOME** | **EFFECT OF 100$** | 0.850 | **0.005** | 0.759 | 0.952 | | 0.753 | **<0.001** | 0.691 | 0.821 |
| **HOUSE CONSTR** | **CEM/BR vs. BAMB** | 0.622 | **<0.001** | 0.484 | 0.799 | | 0.513 | **<0.001** | 0.413 | 0.637 |
| **MATERNAL GOODS** | **3-4 vs. 0-2** | 0.665 | **<0.001** | 0.534 | 0.827 | | 0.536 | **<0.001** | 0.441 | 0.650 |
| **POT WATER** | **Y vs. N** | 1.109 | 0.355 | 0.891 | 1.381 | | 1.173 | 0.112 | 0.964 | 1.427 |
| **BATHROOM** | **Y vs. N (time-vary)** | 0.719 | **<0.001** | 0.600 | 0.861 | | 0.632 | **<0.001** | 0.497 | 0.804 |
| **DOG IN HOUSE** | **Y vs. N** | 1.014 | 0.923 | 0.764 | 1.346 | | 1.274 | 0.083 | 0.969 | 1.676 |
| **CAT IN HOUSE** | **Y vs. N** | 1.122 | 0.511 | 0.796 | 1.581 | | 1.118 | 0.399 | 0.863 | 1.448 |
| **PIGS** | **Y vs. N (time-vary)** | 1.056 | 0.613 | 0.855 | 1.305 | | 1.050 | 0.721 | 0.802 | 1.376 |
| **AGR EXP** | **Y vs. N** | 0.865 | 0.213 | 0.689 | 1.087 | | 0.923 | 0.411 | 0.762 | 1.117 |
| **MATERNAL STH** | **Y vs. N** | 3.105 | **<0.001** | 2.507 | 3.846 | | 4.217 | **<0.001** | 3.487 | 5.101 |
| **PATERN STH** | **Y vs. N** | 2.370 | **<0.001** | 1.637 | 3.433 | | 3.312 | **<0.001** | 2.415 | 4.542 |
| **ANY STH** | **Y vs. N** | 2.721 | **<0.001** | 2.134 | 3.471 | | 4.009 | **<0.001** | 3.210 | 5.007 |
| **ANY (EXCL. PARENTS) STH** | **Y vs. N** | 3.015 | **<0.001** | 2.306 | 3.942 | | 3.524 | **<0.001** | 2.745 | 4.526 |
| **SIBLINGS STH** | **Y vs. N** | 3.315 | **<0.001** | 2.472 | 4.446 | | 4.156 | **<0.001** | 3.162 | 5.462 |

S7 Table. Estimates for associations between any childhood soil-transmitted helminth (STH) infection and individual, parental, and household determinants derived using generalized estimation equations (GEE) and Maximum Likelihood Estimates (MLE) for binary longitudinal outcomes under missing completely at random and missing at random assumptions, respectively. GEE provide population average estimates while MLE provide subject-specific estimates.
