# Supplementary material for: The epidemiology of soil-transmitted helminth infections in children up to 8 years of age: Findings from an Ecuadorian birth cohort
Source: PLoS Negl Trop Dis. 2021 Nov 19;15(11):e0009972. doi: 10.1371/journal.pntd.0009972 (PMC8641893; doi:10.1371/journal.pntd.0009972)
Supplement: S8 Table — Complete data analyses from Table 1 are provided for comparison. All ORs were adjusted for age. (DOCX) [file pntd.0009972.s008.docx]

| **ANY CHILDHOOD STH** | | **MISSING OUTCOMES SET TO**  **NEGATIVE** | | | | **MISSING OUTCOME SET TO POSITIVE** | | | | **COMPLETE DATA ANALYSES**  **(from Table 1)** | | | |
| --- | --- | --- | --- | --- | --- | --- | --- | --- | --- | --- | --- | --- | --- |
| **VARIABLE** | **CATEGORY** | **OR** | **p-value** | **95%CI**  **LOW** | **95%CI**  **HIGH** | **OR** | **p-value** | **95%CI**  **LOW** | **95%CI**  **HIGH** | **OR** | **p-value** | **95%CI**  **LOW** | **95%CI**  **HIGH** |
| **GENDER** | **F vs. M** | 1.053 | 0.669 | 0.831 | 1.334 | 0.975 | 0.702 | 0.858 | 1.109 | 1.038 | 0.753 | 0.825 | 1.306 |
| **BIRTH ORDER** | **3-4 vs. 1-2** | 1.419 | **0.001** | 1.156 | 1.743 | 0.958 | 0.560 | 0.828 | 1.107 | **1.396** | **0.003** | **1.119** | **1.741** |
|  | **>=5 vs.1-2** | 2.336 | **<0.001** | 1.700 | 3.210 | 1.352 | **<0.001** | 1.144 | 1.597 | **2.461** | **<0.001** | **1.800** | **3.364** |
| **BREAST FEEDING** | **7-12 vs.0-6** | 0.991 | 0.961 | 0.699 | 1.405 | 0.833 | 0.074 | 0.682 | 1.018 | 0.904 | 0.606 | 0.615 | 1.328 |
|  | **>12 vs.0-6** | 0.878 | 0.413 | 0.643 | 1.199 | 0.838 | 0.068 | 0.693 | 1.013 | 0.803 | 0.234 | 0.559 | 1.153 |
| **DAY CARE 36M** | **Y vs. N** | 1.288 | **0.036** | 1.017 | 1.633 | 1.254 | **0.008** | 1.062 | 1.480 | **1.373** | **0.007** | **1.089** | **1.732** |
| **ANTIPARAS THERAPY** | **Y vs. N (time-vary)** | 0.814 | **0.025** | 0.680 | 0.974 | 1.004 | 0.962 | 0.855 | 1.178 | **0.782** | **0.012** | **0.647** | **0.947** |
| **MATERNAL AGE** | **21-29 vs. <=20** | 1.264 | 0.069 | 0.982 | 1.625 | 0.882 | 0.095 | 0.761 | 1.022 | 1.156 | 0.284 | 0.887 | 1.507 |
|  | **>=30 vs. <=20** | 1.114 | 0.413 | 0.861 | 1.441 | 0.741 | **0.001** | 0.626 | 0.878 | 0.924 | 0.571 | 0.705 | 1.213 |
| **MATERNAL ETHNICITY** | **N-AFRO vs. AFRO** | 0.522 | **<0.001** | 0.419 | 0.649 | 0.949 | 0.454 | 0.826 | 1.089 | **0.512** | **<0.001** | **0.410** | **0.639** |
| **MATERNAL EDU** | **PRIM vs. ILLIT** | 0.544 | **<0.001** | 0.413 | 0.716 | 0.689 | **<0.001** | 0.573 | 0.829 | **0.462** | **<0.001** | **0.345** | **0.619** |
|  | **SECOND vs. ILLIT** | 0.290 | **<0.001** | 0.217 | 0.387 | 0.540 | **<0.001** | 0.444 | 0.659 | **0.229** | **<0.001** | **0.167** | **0.314** |
| **MATERNAL ALLERGY** | **Y vs. N** | 0.687 | 0.095 | 0.442 | 1.068 | 0.926 | 0.507 | 0.736 | 1.163 | 0.706 | 0.131 | 0.449 | 1.110 |
| **MATERNAL ATHOPY** | **Y vs. N** | 0.831 | 0.152 | 0.646 | 1.070 | 0.901 | 0.202 | 0.768 | 1.057 | 0.908 | 0.471 | 0.698 | 1.181 |
| **PATERNAL AGE** | **21-29 vs. <=20** | 0.938 | 0.701 | 0.678 | 1.298 | 0.805 | **0.033** | 0.659 | 0.983 | 0.835 | 0.33 | 0.581 | 1.200 |
|  | **>=30 vs. <=20** | 1.167 | 0.389 | 0.821 | 1.658 | 0.761 | **0.007** | 0.624 | 0.930 | 0.964 | 0.847 | 0.661 | 1.405 |
| **PATERNAL ETHNICITY** | **N-AFRO vs. AFRO** | 0.581 | **<0.001** | 0.466 | 0.725 | 0.918 | 0.255 | 0.793 | 1.064 | **0.567** | **<0.001** | **0.450** | **0.714** |
| **PATERNAL EDUCATION** | **PRIM vs. ILLIT** | 0.543 | **0.001** | 0.377 | 0.782 | 0.818 | 0.058 | 0.665 | 1.007 | **0.517** | **<0.001** | **0.366** | **0.731** |
|  | **SECOND vs. ILLIT** | 0.369 | **<0.001** | 0.247 | 0.551 | 0.739 | **0.005** | 0.598 | 0.914 | **0.364** | **<0.001** | **0.248** | **0.535** |
| **PATERNAL ALLERGY** | **Y vs. N** | 0.593 | 0.167 | 0.282 | 1.245 | 1.440 | **0.017** | 1.068 | 1.942 | 0.670 | 0.205 | 0.360 | 1.246 |
| **PATERNAL ATOPY** | **Y vs. N** | 0.998 | 0.988 | 0.743 | 1.340 | 1.009 | 0.925 | 0.840 | 1.212 | 1.054 | 0.749 | 0.763 | 1.457 |
| **ADMIN RESIDENCE** | **RURAL vs. URBAN** | 1.056 | 0.674 | 0.820 | 1.358 | 0.935 | 0.332 | 0.815 | 1.072 | 0.996 | 0.974 | 0.778 | 1.276 |
| **SOCIO ECON STATUS** | **MED vs. LOW** | 0.776 | 0.094 | 0.577 | 1.045 | 0.967 | 0.678 | 0.826 | 1.132 | **0.750** | **0.04** | **0.570** | **0.988** |
|  | **HIGH vs. LOW** | 0.495 | **<0.001** | 0.391 | 0.626 | 0.772 | **0.001** | 0.663 | 0.898 | **0.463** | **<0.001** | **0.356** | **0.602** |
| **HOUSE CROWDING** | **>=3 vs. <3** | 1.871 | **<0.001** | 1.499 | 2.335 | 1.506 | **<0.001** | 1.327 | 1.709 | **2.096** | **<0.001** | **1.676** | **2.622** |
| **MATERNAL INCOME** | **EFFECT OF 100$** | 0.851 | **0.002** | 0.767 | 0.944 | 0.989 | 0.593 | 0.948 | 1.031 | **0.850** | **0.005** | **0.759** | **0.952** |
| **HOUSE CONSTR** | **CEM/BR vs. BAMB** | 0.664 | **0.001** | 0.521 | 0.845 | 0.975 | 0.745 | 0.834 | 1.139 | **0.622** | **<0.001** | **0.484** | **0.799** |
| **MATERNAL GOODS** | **3-4 vs. 0-2** | 0.727 | **0.004** | 0.585 | 0.904 | 0.776 | **<0.001** | 0.686 | 0.877 | **0.665** | **<0.001** | **0.534** | **0.827** |
| **POTABLE WATER** | **Y vs. N** | 1.051 | 0.646 | 0.851 | 1.297 | 1.109 | 0.120 | 0.973 | 1.265 | 1.109 | 0.355 | 0.891 | 1.381 |
| **BATHROOM TYPE** | **Y vs. N (time-vary)** | 0.641 | **<0.001** | 0.537 | 0.766 | 0.979 | 0.727 | 0.871 | 1.101 | **0.719** | **<0.001** | **0.600** | **0.861** |
| **DOG IN HOUSE** | **Y vs. N** | 0.993 | 0.962 | 0.757 | 1.303 | 0.963 | 0.717 | 0.787 | 1.179 | 1.014 | 0.923 | 0.764 | 1.346 |
| **CAT IN HOUSE** | **Y vs. N** | 1.178 | 0.314 | 0.856 | 1.620 | 0.874 | 0.171 | 0.720 | 1.060 | 1.122 | 0.511 | 0.796 | 1.581 |
| **PIGS** | **Y vs. N (time-vary)** | 1.133 | 0.258 | 0.913 | 1.405 | 0.914 | 0.211 | 0.793 | 1.052 | 1.056 | 0.613 | 0.855 | 1.305 |
| **AGRICULTURE** | **Y vs. N** | 0.998 | 0.990 | 0.795 | 1.254 | 0.787 | **<0.001** | 0.695 | 0.892 | 0.865 | 0.213 | 0.689 | 1.087 |
| **MATERNAL STH** | **Y vs. N** | 2.646 | **<0.001** | 2.111 | 3.316 | 1.675 | **<0.001** | 1.431 | 1.960 | **2.721** | **<0.001** | **2.134** | **3.471** |
| **PATERN STH** | **Y vs. N** | 1.851 | **<0.001** | 1.341 | 2.556 | 1.442 | **0.010** | 1.093 | 1.903 | **3.105** | **<0.001** | **2.507** | **3.846** |
| **ANY STH** | **Y vs. N** | 2.437 | **<0.001** | 1.925 | 3.086 | 1.302 | **<0.001** | 1.131 | 1.499 | **2.370** | **<0.001** | **1.637** | **3.433** |
| **ANY (EXCL. PARENTS) STH** | **Y vs. N** | 2.590 | **<0.001** | 1.993 | 3.365 | 1.389 | **<0.001** | 1.173 | 1.645 | **3.015** | **<0.001** | **2.306** | **3.942** |
| **SIBLINGS STH** | **Y vs. N** | 2.819 | **<0.001** | 2.119 | 3.750 | 1.427 | **<0.001** | 1.170 | 1.741 | **3.315** | **<0.001** | **2.472** | **4.446** |

S8 Table. Sensitivity analyses for associations between any childhood soil-transmitted helminth (STH) infections and individual, parental, and household determinants derived using generalized estimation equations (GEE). Complete data analyses from Table 1 are provided for comparison. All ORs were adjusted for age.
