# Supplementary material for: The epidemiology of soil-transmitted helminth infections in children up to 8 years of age: Findings from an Ecuadorian birth cohort
Source: PLoS Negl Trop Dis. 2021 Nov 19;15(11):e0009972. doi: 10.1371/journal.pntd.0009972 (PMC8641893; doi:10.1371/journal.pntd.0009972)
Supplement: Attachment — Submitted filename: Point-by-point response_PLoS_NTD_17_09_21.docx [file pntd.0009972.s010.docx]

**Point-by-point responses to reviewer's questions**

Key Review Criteria Required for Acceptance?

As you describe the new analyses required for acceptance, please consider the following:

Methods
-Are the objectives of the study clearly articulated with a clear testable hypothesis stated?
-Is the study design appropriate to address the stated objectives?
-Is the population clearly described and appropriate for the hypothesis being tested?
-Is the sample size sufficient to ensure adequate power to address the hypothesis being tested?
-Were correct statistical analysis used to support conclusions?
-Are there concerns about ethical or regulatory requirements being met?

Reviewer #1: Yes

Reviewer #2: This MS describes a very impressive prospective study, an approach that is rare as highlighted by the authors. Particular strengths include the length of the longitudinal study, the large sample size, the fact that 4 diagnostic methods were used and the complex statistical analyses employed.

Response 1: We would like to thank both reviewers for their positive reviews of our manuscript.

Results
-Does the analysis presented match the analysis plan?
-Are the results clearly and completely presented?
-Are the figures (Tables, Images) of sufficient quality for clarity?

Reviewer #1: Yes

Reviewer #2: My comments are minor and mainly relate to some more explanation of the statistical analyses.

Response 2: Thank you. We have made changes and corrections to the manuscript which we hope provides greater clarity.

Abstract
Line 37: “Peaked in risk” sounds a little clumsy to me

Response 3: The sentence in the Abstract (lines 36-37) has changed to: “The risk of *A. lumbricoides* (16.2%) was greatest at 3 years, while risks of any STH (25.1%) and *T. trichiura* (16.5%) peaked at 5 years.”

Methods
I would like to see a little explanation as to why the authors decided not use intensity (e.p.g) as a continuous variable in their analyses?

Response 4: Infection intensities with *A. lumbricoides* and *T. trichiura* exhibited large variabilities and an excessive number of zeros. Therefore, the distributions of e.p.g for *A. lumbricoides* and *T. trichiura* were highly skewed even after log(x+1) type transformation with excessive zeros, violating key assumptions for longitudinal regression models. We have now done additional analyses, namely zero-inflated type models fitted to the count outcomes. We have fit a zero inflated Poisson model as well as a zero inflated Negative Binomial model accounting for the individual longitudinal cluster. As the latter was a better fit as indicated by AIC and BIC criteria, only these results are presented in the Supplementary Tables S5 and S6 for each of A. lumbricoides and *T. trichiura*, respectively. A zero-inflated model assumes that the zero count in the outcome is due to two different processes. For instance, an individual participant can test either negative or positive. If negative, the only outcome possible is zero. If positive, the count is represented by a negative binomial process and the zeros corresponding to this process indicate very small counts which may be undetectable by the diagnostic methods used.

The definite zeros are modelled by a logistic regression, the counts are modelled by the negative binomial model. The resulting estimates consist of odds ratios (transformed such that they indicate association with “not being zero” to relate their consistency with the binary outcomes results) and relative ratios indicating a fold change (or decrease) in intensity with the variables of interest. The first set of estimates (ORs) of the Supplementary Tables S5 and S6 are remarkably similar in magnitude and precision with the findings for the binary outcome (i.e. positive vs. negative) shown in Supplementary Tables S3 and S4, respectively. For this reason, we felt such models would be more difficult to explain and digest for a wider audience and, for simplicity and sake of simple communication we chose to present the binary outcomes. Moreover, goodness of fit for these models, i.e. comparison between raw observations and predicted values, is difficult to assess as it is problematic to disentangle which zeros are modelled by logit and which by the negative binomial part in the raw data.

We have added appropriate explanatory text in Methods (lines 199-205) and Results (lines 349-352) as follows:

“Distributions of infection intensities (epg) for *A. lumbricoides* and *T. trichiura were* highly skewed and analyzed using zero-inflated models [17] assuming two sources of zeros: those partly belonging to the count distribution and resulting from undetectable infections and those partly representing a negative test. Estimates of effect derived from these analyses indicated associations of “non-zero occurrences” (provided as ORs) or fold-change (in epg) with covariates. All estimates were age-adjusted and accounted for the hierarchical structure of outcomes.”

“Associations of infection intensities with *A. lumbricoides* and *T. trichiura* in age-adjusted models are shown in Tables S5 and S6, respectively: findings for both infections were consistent with those of analyses for binary outcomes”.

Line 157: I would like to see an explanation of “two level logistic regression”

Response 5: The outcomes of these analyses of the baseline cohort are of binary nature indicating presence/absence of the infection in the individual. Applying a simple or one-level logistic regression would require observations to be independent. These data exhibit hierarchical features with individuals (level 2) belonging to the same household (cluster – level 1) meaning that the observations are not independent and therefore violating a fundamental assumption for the applicability of a (one-level) logistic regression. Failing to account for the two sources of variability in the data - that between households and within individuals’ households - would result in incorrect inferences (i.e. wrong p-values and standard errors). Belonging to the same household creates a level of dependencies between participants which needs to be accounted for in the analysis.

The methodology is well known in the literature and is one of the simplest settings for hierarchical (clustered) binary data. A comprehensive read on the methodology and rationale can be found in Reference 11 (Goldstein H. Multilevel statistical models. 4th ed. Oxford: Wiley; 2011). We have modified the text in the Statistical analysis section of Methods as follows (lines 155-163):

“To estimate age-dependent risk of STH infections around the time of birth of cohort children, using data from stool samples collected from members of cohort households that have a clustered hierarchical structure (defined by households and individual participants), we used two-level logistic regression (households at the first and participants within households at the second levels) [11] to account for intra- and inter-household variability. Estimates were based on 6800 stool samples collected from 1973 households with a median size of 3 members (interquartile range, 1-5). The approach produced adequate associated standard errors which accounted for the dependencies in the data attributable to participants living within the same household.”

Line 170: Binary random effects models were also considered – with what decision?

Response 6: The choice of modelling is dictated by the nature of the outcome variables (longitudinal binary in our settings) and by the study questions. There are two main approaches in the literature, extensively discussed in References 12 (Fitzmaurice G, Davidian M, Verbeke G, Molenberghs G. Longitudinal data analysis. 1st ed. Boca Raton: Chapman and Hall/CRC; 2008) and 13 (Szmaragd C, Clarke P, Steele F. Subject specific (SS) and population average (PA) models for binary longitudinal data: a tutorial. Longitudinal and Life Course Studies. 2013;4(2):147-165). These two approaches represent two basic types of longitudinal methods analysis using ‘population average’ and ‘subject-specific’ models that can differ for longitudinal binary outcomes. The differences stem from the estimation methods (generalized estimating equations referred to as GEE vs. maximum likelihood estimates referred to as MLE) and from assumptions made on the missing data due to attrition – a common feature of such studies. There are also differences in the interpretation of the estimates: the resulting OR following fitting of PA models measures the change in OR for average change in the explanatory variable across all individuals, while the resulting OR following fitting of SS models measures the change in OR associated with individual change in the explanatory variable. Hence, the latter is particularly valuable for time-varying covariates. Our questions are addressed at the population level hence our preference for PA models. Nevertheless, Table S7 in the supplementary material shows both sets of estimates and reveal differences as might be expected although providing a similar epidemiological interpretation. The PA and SS estimates and their uncertainties are of importance for researchers interested in mathematical modelling aiming at assessing and comparing the effect of various measures to derive the optimal strategies for disease control. The relevant paragraph of the revised text of the manuscript reads as follows (lines 174-179):

“The latter was particularly appropriate for time-varying exposures (i.e. bathroom type, anthelmintic treatments, and household pigs) and can indicate associations of outcome with individual changes in these exposures during follow-up. We used the assumption of an unstructured correlation structure [11-13] and, for the sake of simplicity and given that our questions were addressed at the population level, we have presented and commented on population average estimates only.”

Line 188: Patterns in missing data analyses and sensitivity analyses – again what was the outcome of these analyses? Could the results in the supplementary table be summarised in some way in the main body of the MS to inform readers more easily?  I know you mention this in the Discussion but I think a little more detail is required.

Response 7: Supplementary Table S8 presents estimates for extreme case scenarios in which missing observations in the STH outcome are set to either positive or negative (displayed side by side with estimates shown in Table 1). The relative closeness of the estimates and their overlapping 95% CIs are consistent with robustness of the estimates. We have added further details on the outcome of these analyses to the text of Results as follows (lines 361-368).

“Sensitivity analyses using extreme values for missing data for any STH infections over the period of follow-up (i.e. one in which the missing responses were either considered all negative or all positive) generally preserved the sense of associations observed for analyses using complete data (Table S8): age-adjusted estimates for these extreme scenarios are displayed beside complete data estimates in Table S8. Estimates from these extreme value analyses were comparable to complete data estimates (i.e. 95% CIs intersected) indicating robustness of our findings.”

Line 201: What is “minor” assent?

Response 8: Informed written consent cannot be legally provided by a minor (i.e. aged below 18 years). Informed written consent in this study was provided by the child’s parents or legal guardian. Minor assent refers to agreement to participate in a research study by a legal minor. Minor assent was obtained after informed written parental consent, by explaining the objectives and procedures of the study in a way that would be comprehensible to a child of 8 years in the study setting and in the presence of the child’s parents. If the child agreed to participate, the minor assent form was read to the child by the investigator (in the presence of the child’s parents) and asked if she/he had any questions about the study. Having addressed any questions about the study, the assent form was then given to the child to review and sign (write name) if she/he was in agreement to participate.

--------------------

 Conclusions:
-Are the conclusions supported by the data presented?
-Are the limitations of analysis clearly described?
-Do the authors discuss how these data can be helpful to advance our understanding of the topic under study?
-Is public health relevance addressed?

Reviewer #1: Yes

Reviewer #2: Figure 2: There are interesting differences in the degree of infection between mothers and fathers. Could you provide possible explanations?

Response 9: Prevalence of infection with any STH and each of the two dominant parasites, *A. lumbricoides* and *T. trichiura*, was greater in mothers than fathers. Stool samples were collected from mothers during the third trimester of pregnancy (lines 122-123). An apparent increased risk of STH infection in mothers might be explained by their close contact with young children (Van Eijk et al, PLoS Negl Trop Dis; 2009; 3: e370) geophagy (Luoba et al, Tropical Med Int Health 2005;10:220-7), or by an increased susceptibility to infection relating to immunological changes associated with gestation (Adegnika et al, Wien Klin Wochenschr 2007;119:712-6). A formal evaluation of whether pregnancy per se affects susceptibility to STH infection would require a comparison group of non-pregnant women of reproductive age as has been done previously (Adegnika et al, 2007).

It is very interesting to me that you observe differences in Ascaris and Trichuris as too often, these two parasites are conflated epidemiologically. Can you address the issue of their differences a little more in your Discussion for example differences egg survival, transmission pattern etc?

Response 10: We agree that shared risk factors often lead to an epidemiological conflation of infections with the two parasites. Focusing on the findings of multivariable analyses in Supplementary Tables S3 and S4, some risk factors were shared for both parasites (e.g. age, ethnicity, household overcrowding) while low (versus high) socioeconomic status and having infected siblings appeared to be more strongly related to *T. trichiura*. An association between *A. lumbricoides* and recent anthelmintic treatments was seen in age-adjusted but not multivariable analyses. *A. lumbricoides* eggs appear to be more resilient than *T. trichiura* (Bundy and Cooper,1989) and embryonate more rapidly in the environment which likely favours transmission (Brooker et al, 2006). Sharing a poor household with infected siblings (who frequently contaminate the household environment with *T. trichiura* eggs) may provide a sufficient density of embryonated eggs in the immediate household environment to maximize the chance of successful transmission to a younger uninfected sibling, while *A. lumbricoides* may be less dependent on such conditions for transmission. A study of an indigenous population in Panama showed spatial clustering of *T. trichiura* but not *A. lumbricoides* infection to areas with households with greatest levels of poverty (Halpenny et al, 2013). We have added the following text to the Discussion in response to this and later comments (lines 465-479):

“Infections with *A. lumbricoides* were observed more frequently than *T. trichiura* at an earlier age but by 2 years frequencies of the two parasite species were similar, an observation also seen in a previous longitudinal study of infants in Iquitos, Peru [7]. It is possible that *A. lumbricoides* eggs - that appear to be more resilient, sticky, embryonate more rapidly, and can survive for years [24,25] - may be more uniformly distributed in the environment increasing risk of infection in infants [26]. Opportunities for ingestion of embryonated *T. trichiura* eggs may increase as children become older and start to explore the environment beyond the confines of the immediate household. We observed strong associations between *T. trichiura* infections and living in the poorest households and with infected siblings. Sharing a poor household with infected siblings, who might be expected to contaminate frequently the peri-domestic environment with *T. trichiura* eggs, could maximize the chance of transmission to younger siblings. An analysis in an indigenous population in Panama showed spatial clustering of *T. trichiura*, but not *A. lumbricoides*, to areas with households with the greatest levels of poverty [26].”

--------------------

Editorial and Data Presentation Modifications?
Use this section for editorial suggestions as well as relatively minor modifications of existing data that would enhance clarity. If the only modifications needed are minor and/or editorial, you may wish to recommend “Minor Revision” or “Accept”.

Reviewer #1: None

Reviewer #2: See above
Minor revision only

--------------------

Summary and General Comments:
Use  this section to provide overall comments, discuss strengths/weaknesses of the study, novelty, significance, general execution and scholarship. You may also include additional comments for the author, including concerns about dual publication, research ethics, or publication ethics. If requesting major revision, please articulate the new experiments that are needed.

Reviewer #1: The work by Ster et al focusses on a large Ecuadorian birth cohort followed from birth to the age of 8 in the context of soil transmitted helminths. As far as I am aware, this is a pretty unique cohort in terms of the length of study (8 years), the data from which offers to identify risk factors for STH infections. This sort of data is of immense value to the research community and the comparisons made range across for example age, day-care, educational level, and infection in other household members. The authors conclude, perhaps not surprisingly, that STH infections among the house hold members is a key risk factor; but there are also other important gains of knowledge within this manuscript too.

Some interesting differences in the context of risk factors for having Ascaris versus Trichuris are identified, which would be worth discussing. For example:
Why would having a WC inversely associate with Ascaris but not whipworm?

Response 11: As discussed in responds 10, the findings of multivariable analyses for risk factors associated with *A. lumbricoides* and *T. trichiura* (Supplementary Tables S3 and S4) showed that low socioeconomic status (versus high) and having infected siblings were significantly associated with *T. trichiura* but not *A. lumbricoides*. Having a WC was inversely associated in age-adjusted but not multivariable analyses, and the effect seemed to be explained by other variables. The effect of a WC in the house vs. latrine was more prominent for the combined STH outcome (Table 1) that remained statistically significant in the multivariable model.

Does the lack of an inverse association between whipworm and drug treatment infer drug resistance within Trichuris parasite population?

Response 12: There seemed to be an inverse association between anthelmintic treatments and *A. lumbricoides* but not *T. trichiura* in the age-adjusted models. The effect disappeared in the multivariable model for *A. lumbricoides*. The most likely explanation for a possible stronger effect of anthelmintic treatment (single dose 400 mg albendazole for children aged 2 years or older (lines 213-216) would is the much greater efficacy of this treatment against *A. lumbricoides*: cure rates with 400 mg albendazole are approximately 32% for T. trichiura versus 97% for A. lumbricoides (Else et al, 2020).

Why do Ascaris infections emerge first before whipworm?

Response 13: We agree that infections with *A. lumbricoides* were more frequent than with *T. trichiura* from 7 months to 2 years of age or so (Figures 3-5). This observation is consistent with a previous longitudinal study done in Iquitos, Peru (Reference 7). One possible explanation is that *A. lumbricoides* eggs - that appear to be more resilient, sticky, and can survive for years (Bundy and Cooper, 1989; Brooker et al, 2006) - may be more uniformly distributed in the environment increasing the risk of infection in less mobile infants. Likelihood of ingestion of embryonated *T. trichiura* eggs may be more dependent on exposures to microniches that favour T*. trichiura* egg densities and survival to which an older child may become exposed as they start to explore the peri-domestic environment beyond the confines of the immediate household.

We have added text to the Discussion as follows (lines 454-468):

“Infections with *A. lumbricoides* were observed more frequently than *T. trichiura* at an earlier age but by 2 years frequencies of the two parasite species were similar, an observation also seen in a previous longitudinal study of infants in Iquitos, Peru [7]. It is possible that *A. lumbricoides* eggs - that appear to be more resilient, sticky, embryonate more rapidly, and can survive for years [24,25] - may be more uniformly distributed in the environment increasing risk of infection in infants [26]. Opportunities for ingestion of embryonated *T. trichiura* eggs may increase as children become older and start to explore the environment beyond the confines of the immediate household. We observed strong associations between *T. trichiura* infections and living in the poorest households and with infected siblings. Sharing a poor household with infected siblings, who might be expected to contaminate frequently the peri-domestic environment with *T. trichiura* eggs, could maximize the chance of transmission to younger siblings. An analysis in an indigenous population in Panama showed spatial clustering of *T. trichiura*, but not *A. lumbricoides*, to areas with households with the greatest levels of poverty [26].”

Why for Trichuris does having infected sibling matter so much more re risk of subsequent STH infection?

Response 14: Please see responses 10 and 13 above.

Further would it also be possible from the data sets to analyse whether attendance at school altered risk? (at what age do children start attending school?)

Response 15: Children attend school in Ecuador from 5-6 years. To our knowledge all cohort children were school attenders although we did not collect specific information to determine this.

Overall the manuscript provides new important knowledge which will be of interest to the readers of PLOS NTDs

Reviewer #2: (No Response)

--------------------

PLOS authors have the option to publish the peer review history of their article (<a href="<https://journals.plos.org/plosntds/s/editorial-and-peer-review-process#loc-peer-review-history>" target="_blank">what does this mean?). If published, this will include your full peer review and any attached files.

If you choose “no”, your identity will remain anonymous but your review may still be made public.

Do you want your identity to be public for this peer review? For information about this choice, including consent withdrawal, please see our href="<https://www.plos.org/privacy-policy>" target="_blank">Privacy Policy.

Reviewer #1: Yes: Kathryn Else

Reviewer #2: No

Figure Files:
While revising your submission, please upload your figure files to the Preflight Analysis and Conversion Engine (PACE) digital diagnostic tool, <https://pacev2.apexcovantage.com>. PACE helps ensure that figures meet PLOS requirements. To use PACE, you must first register as a user. Then, login and navigate to the UPLOAD tab, where you will find detailed instructions on how to use the tool. If you encounter any issues or have any questions when using PACE, please email us at [figures@plos.org](mailto:figures@plos.org).
 
Data Requirements:
Please note that, as a condition of publication, PLOS' data policy requires that you make available all data used to draw the conclusions outlined in your manuscript. Data must be deposited in an appropriate repository, included within the body of the manuscript, or uploaded as supporting information. This includes all numerical values that were used to generate graphs, histograms etc.. For an example see here: <http://www.plosbiology.org/article/info%3Adoi%2F10.1371%2Fjournal.pbio.1001908#s5>.
 
Reproducibility:
To enhance the reproducibility of your results, we recommend that you deposit your laboratory protocols in protocols.io, where a protocol can be assigned its own identifier (DOI) such that it can be cited independently in the future. Additionally, PLOS ONE offers an option to publish peer-reviewed clinical study protocols. Read more information on sharing protocols at <https://plos.org/protocols?utm_medium=editorial-email&utm_source=authorletters&utm_campaign=protocols>

References
Please review your reference list to ensure that it is complete and correct. If you have cited papers that have been retracted, please include the rationale for doing so in the manuscript text, or remove these references and replace them with relevant current references. Any changes to the reference list should be mentioned in the rebuttal letter that accompanies your revised manuscript. If you need to cite a retracted article, indicate the article's retracted status in the References list and also include a citation and full reference for the retraction notice.

__________________________________________________
In compliance with data protection regulations, you may request that we remove your personal registration details at any time.  (Use the following URL: <https://www.editorialmanager.com/pntd/login.asp?a=r>). Please contact the publication office if you have any questions.
